# Detection of hepatitis B virus DNA among accepted blood donors in Nanjing, China

**DOI:** 10.1186/1743-422X-7-193

**Published:** 2010-08-19

**Authors:** Yong Liu, Ping Li, Cuiping Li, Jinyong Zhou, Chao Wu, Yi-Hua Zhou

**Affiliations:** 1 Department of Laboratory Medicine, Nanjing Drum Tower Hospital, Nanjing University Medical School, Nanjing, China; 2 Department of Transfusion Medicine, Nanjing Drum Tower Hospital, Nanjing University Medical School, Nanjing, China; 3 Department of Infectious Diseases, Nanjing Drum Tower Hospital, Nanjing University Medical School, Nanjing, China; 4 Jiangsu Key Laboratory for Molecular Medicine, Nanjing University Medical School, Nanjing, China

## Abstract

**Background:**

Posttransfusion hepatitis B virus (HBV) infection still occurs although its incidence has been substantially reduced since the introduction of screening of hepatitis B surface antigen (HBsAg) in blood donors. This study aimed to investigate the occult HBV infection in accepted blood donors in Nanjing, China.

**Results:**

The lower detection limit of the nested PCR in this study was estimated to be 20 copies/ml HBV DNA. The positive rate of occult HBV infection was 0.13% (5 of 2972) in the accepted blood donors. Sequencing data showed that the amplified HBV sequences were not identical each other and to the known sequences cloned in our laboratory, excluding the false-positive caused by cross-contamination. Phylogenetic analysis showed that the HBV in all five donors was genotype B; a single base deletion was detected in the S region of HBV DNA from one donor, and no mutation was observed in the "a" determinant of HBsAg from four other donors. All five donors were negative for anti-HBs and one was positive for anti-HBc.

**Conclusions:**

The prevalence of occult HBV infection in the accepted blood donors in Nanjing, China is relatively high. The data would be meaningful in adapting strategy to eliminate posttransfusion HBV infection in China.

## Background

Hepatitis B virus (HBV) infection is one of the major health problems worldwide. The infection is usually defined by the presence of hepatitis B surface antigen (HBsAg) in serum or plasma. However, HBV may exist in humans without detectable HBsAg but with presence of HBV DNA in the serum and/or in the liver, i.e. the occult HBV infection [1]. The occult infection may result from the low viral load in circulation (usually <200 IU/ml) [2] or a mutant HBsAg which is not recognized by the monoclonal antibodies against HBsAg (anti-HBs) used in some commercial detection kits [1,3].

Because of routine screening of blood donors for HBsAg, the incidence of transfusion-transmitted hepatitis B has been steadily reduced over the last four decades; however HBV transmission remains the most frequent transfusion-transmitted viral infection [4-6]. The residual risk of HBV transmission by transfusion is mainly associated with occult HBV infection in blood donors. Additionally, occult HBV infection also has significance in bone marrow and organ transplantations [2,7-9].

Attributed to widespread use of hepatitis B vaccine and other strategies for control of HBV infection, the prevalence of HBsAg carrier rate in general population in China decreased from some 10.0% in 1980 s to 7.2% in 2006 [10-12]. However, HBV infection is still endemic in China. Studies have shown that the prevalence of occult HBV infection is closely related to the endemicity of HBV infection [13,14], however, the occult HBV infection in China has been less studied [15-17]. Thus, we performed this study to investigate the prevalence of occult HBV infection in blood donors in Nanjing, an eastern region of China.

## Results

### The sensitivity of the nested PCR

**Figure 1:**
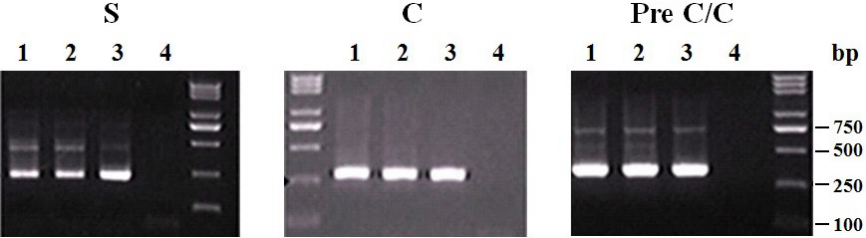
**The lower detection limit of the nested PCR**. Lanes 1-4, HBV positive control with 2000, 200, 20, and 2 copies/ml respectively.

To determine the sensitivity of the nested PCR developed in this study, we performed the PCR using 10-fold serially diluted template DNA extracted from a plasma with a concentration of 2000 copies/ml HBV DNA. As shown in Figure 1, the minimum copies detected by the three sets of the primers were all 20 copies/ml HBV DNA, equivalent to 1 copy per reaction. Thus, the lower detection limit of the nested PCR using the different primers was comparable.

### Positive rate of HBV DNA in accepted blood donors

The samples in which HBV DNA was detected by at least two sets of the primers were considered to be positive for HBV. Of the 2972 accepted blood donors, 5 (0.17%) were determined to have occult HBV infection (Table 1). The plasmas of the nested PCR positive samples were also measured by fluorescent real-time PCR, and the results showed that the viral load in each sample was less than 500 copies/ml. However, when the three plasmas (200 μl for each) were subjected to DNA extraction and the extracted DNA was dissolved in 20 μl TE buffer, HBV DNA became detectable by the fluorescent quantitation PCR with 2.1 × 10^3 ^and 1.2 × 10^4 ^copies/ml respectively. Compared with the final volume of 40 μl DNA extracted from 20 μl plasmas according to the instruction of the real-time PCR kit, the DNA extraction steps in this study actually concentrated the plasma DNA with 20-fold. Thus, by reverting the concentrated DNA to the original level, the actual viral loads in these two plasmas were approximately 105, 110, and 600 copies/ml respectively (Table 1).

**Table 1:** Laboratory data of donors with occult HBV infection^a^

Sample ID	Primers			Viral load^b ^(copies/ml)	Anti-HBc
			
	S	Pre C/C	C		
471	P	ND	P	ND	N
686	P	ND	P	ND	N
878	P	ND	P	110	P
1473	P	P	N	105	N
1475	P	P	P	600	N

ND: not done

N: negative

P: positive

^a^All five donors were negative for HBsAg and anti-HBs.

^b^DNA was extracted from 200 μl plasma by phenol-chloroform.

Of the five HBV DNA positive samples, one was positive for anti-HBc and four others were negative, and all of them were negative for anti-HBs. The ALT of all the positive samples was normal (Table 1).

### HBV sequencing and genotyping

**Figure 2:**
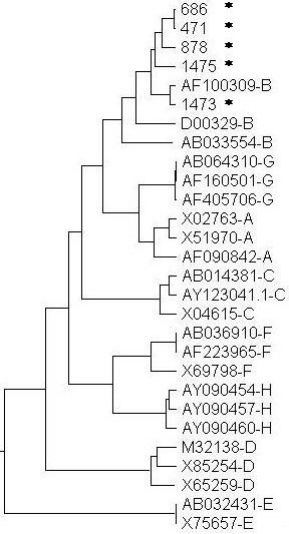
**Phylogenetic analysis of S region of HBV**. Phylogenetic tree of the partial S gene sequences (185 bp) from five donors with occult HBV infection in this study and sequences recovered from GenBank. Sequences retrieved from GenBank are denoted by their accession numbers and genotype. *, S gene sequences obtained in this study.

The PCR products amplified by the S region primers were sequenced; the readable sequences were more than 180 bps and contained the sequences of the "a" determinant of HBsAg. Sequencing data demonstrated that none of them was identical to the known sequences detected in our laboratory and there were at least 2 bps differences among the different plasmas, excluding the false positive caused by cross-contamination. A single base deletion was detected in the S region from a donor, and no mutation in the "a" determinant was observed in four other donors after comparing with the known wild sequences in the GenBank. Phylogenetic analysis of the partial S-gene sequences from the five samples and corresponding sequences recovered from GenBank demonstrated that the viruses in the five samples all belonged to genotype B (Figure 2).

## Discussion

We investigated the occult HBV infection by detecting HBV DNA in plasmas from accepted blood donors in China with nested PCR and found that the positive rate of HBV DNA was 0.17% (5/2972). Compared with the risk of posttransfusion HBV infection in countries in which nucleic acid amplification tests (NAT) are used to screen HBV in blood donors [18,19], the data in the present study indicate that there is still substantial risk for posttransfusion HBV infection in China despite screening for HBsAg in blood donors.

Although nested PCR is sometimes associated with the risk of false-positive results, the HBV DNA detected in this study is unlikely to be caused by cross-contamination or carryover because of the following reasons: (1) we took particular precautions in performing each PCR step as suggested by Kwok and Higuchi [20]; (2) the DNA extraction and each PCR round were undertaken in different rooms; (3) the positive samples were separately tested with two different sets of primers and retested by real-time PCR; and (4) most importantly, the sequencing data showed that all sequences of the positive samples were not identical to each other and to the sequences amplified in our laboratory. On the other hand, the nested PCR used in this study was highly sensitive as it detected as low as 20 copies/ml HBV DNA (Figure 1). Although detection of HBV DNA using liver tissue DNA extracts can increase the positive rate of occult HBV infection [21], it is not feasible to get liver tissues from the blood donors. Thus, under the current circumstance and with the available techniques, the positive rate of occult HBV infection detected in this study is less likely to be underestimated.

The prevalence of occult HBV infection in blood donors appears to be varied in different countries or even in different areas in a country. The variations may be associated with the different assays used in the various surveys and the HBV endemicity. It has been documented that low, intermediate, or high endemic areas have different occult HBV infection rate; the NAT tests in blood donors demonstrated that occult HBV infection in regions with low HBV endemicity, such as Western countries is rare, whereas in Japan and Hong Kong it is much higher [22]. Indeed, the occult HBV infection rate (0.17%) in blood donors in the present study is higher than that in Western countries like Canada and Germany [23,24], and comparable to that in Ghana [25].

In China, a few relevant studies show substantially divergent prevalence of occult HBV infection in the blood donors, ranging from 0.028% (8/28800) in Guangdong Province [26] and 2.71% (18/1698) in Zhejiang Province [27] to as high as 9.78% (57/583) in Hunan Province [28]. The relatively low rate in Guangdong may be due to the mini-pool (24 samples) approach used in that study. In the present study, we used real-time PCR to detect HBV DNA in all five plasmas with occult HBV infection, but none of them had a detectable level at a lower detection limit of 500 copies/ml. On the other hand, the high frequency of 9.8% found among accepted blood donors in Hunan [28] is questionable, since that report did not exclude the possibility of cross-contaminations.

Several factors are involved in the recognition of the occult HBV infection, including viral variants carrying mutant HBsAg that are not recognized by specific antibodies used in assays for HBsAg [29-32] and low level expression of HBV genes [33-35]. In the present study, the mutation in the "a" determinant of S region was observed in only one donor and there was no mutation in the "a" determinant in four other donors, yet their HBV DNA levels were only between 105 and 600 copies/ml. Thus, strong suppression of viral replication and gene expression may have resulted from the host immune responses [36]. Alternatively, since four of the five occult infections did not show any serological HBV markers, it is also possible that these samples were collected during the seroconversion window period, in which case further follow-up may have identified this serologic phenomenon [37]. However, this issue can not be clarified since we did not follow-up these blood donors.

The occurrence of posttransfusion hepatitis B in China has been substantially reduced since the introduction of HBsAg screening of blood donors. However, the true incidence remains unknown because of the obstacles in conducting such studies. In the present survey, we found that 0.17% of the accepted blood donors had occult HBV infection, which is much higher than that observed in Western counties as well as in Japan and Hong Kong. Although the conversion of residual risk of occult HBV infection into the true rate of infection is largely unknown, the results in the chimpanzee model demonstrate that as low as 10 copies of HBV may result in half of the animals being infected, irrespective of HBV genotype [38,39]. Considering that our blood donors had HBV DNA exceed 100 copies/ml, the recipients of these five occult HBV carrier donors were highly possible to be infected with HBV although we did not follow-up the recipients. Indeed, numerous reports have documented that occult HBV infection in blood donors may cause posttransfusion hepatitis B [40-42].

Currently, only a few financially prosperous cities in China detect HBV DNA in blood donors by minipool NAT. We consider that NAT for HBV DNA as a routine screening in blood donors in China will be cost-effectiveness, since the risk of HBV transmission by transfusion in China is relatively high based on our data. Even in countries with low HBsAg prevalence such as USA and some European countries, minipool NAT has been used as a routine test in blood donors [43].

## Conclusions

The data in the present study will be meaningful in setting up a strategy to prevent posttransfusion hepatitis B in China. Because of relatively high rate of occult HBV infection in blood donors in China, tests for HBV DNA in blood donors would substantially reduce the incidence of posttransfusion hepatitis B.

## Methods

### Blood donors

We randomly collected plasma samples from 2972 accepted volunteer donors at the Nanjing Red Cross Blood Center between February 2007 and April 2008. Each sample was not directly collected from the donor, but collected from one tubing segment connected to the bag of the donor's blood; each tubing segment was cut with a fresh blade to avoid cross-contamination among samples. According to the policy set up by the China Health Ministry, an accepted donor is defined as a generally healthy person with normal alanine aminotransferase (ALT), negative for HBsAg and antibodies against hepatitis C virus (HCV), HIV, and treponema pallidum. Thus, the laboratory tests in all the 2972 donors showed to be normal ALT and negative for HBsAg, anti-HCV, anti-HIV, and antibody against treponema pallidum; the HBsAg in each donor was tested in parallel by two different commercial enzyme-linked immunosorbent assay (ELISA) kits. The donors' average age was 24.9 years (range, 18-53). They were 1475 males and 1497 females. The plasmas were stored at -20°C. All the experiments were approved by the Ethics Committee of Nanjing Drum Tower Hospital, Nanjing University Medical School, in accordance with guidelines of the Nation Health and Medical Research Council of China.

### Plasma DNA isolation

DNA was extracted from plasma using phenol-chloroform extraction by the standard method. Briefly, 200 μl plasma was treated with proteinase K at 50°C for 3 h, and then DNA was extracted twice by phenol-chloroform and rinsed once by 70% ethanol. Finally, 20 μl Tris-EDTA (TE) buffer was added to each sample to dissolve DNA.

### Detection of HBV-DNA by nested PCR

Nested PCR was performed using specific primers derived from the regions coding for HBsAg, hepatitis B core antigen (HBcAg) and pre-C respectively as previously reported with minor modifications [44-46]. Primer sequences are shown in Table 2. In brief, the first round PCR was carried out in a final volume of 50 μl containing 1.25 units of Taq polymerase (Takara, Dalian, China) and 5 μl DNA template. The amplification was carried out for 35 cycles (20 seconds at 94°C, 30 seconds at 55°C, 45 seconds at 72°C) after initial denaturation for 2 min. A final extension step was performed for 10 min at 72°C. The second round PCR was carried out using 5 μl of the first PCR product under the same condition as the first round PCR except 25 pmol of each internal primer was used. The PCR products were electrophoresed on 1.5% agarose gel and stained with ethidium bromide, and then photographed by image analysis system (UVP, Upland, CA).

**Table 2:** Primers used for nested PCR

Region	Name	Sequences 5' to 3'	Amplicon size (bp)
S	1stS192F	TCGTGTTACAGGCGGGGTTT	513
	1stS704R	CGAACCACTGAACAAATGGC	
	2ndS455F	CAAGGTATGTTGCCCGTTTG	233
	2ndS687R	GGCACTAGTAAACTGAGCCA	

C	1stC1869F	ACTGTTCAAGCCTCCAAGCT	600
	1stC2468R	GGAATACTAACATTGAGATTCCCGAG	
	2ndC2015F	TGCTCTGTATCGGGAGGC	280
	2ndC2294R	AGTGCGAATCCACACTC	

Pre C/C	1st 2023F	GCCTTAGAGTCTCCTGAGCA	442
	1st 2464R	GTCCAAGGAATACTAAC	
	2nd 2046F	CCTCACCATACTGCACTCA	340
	2nd 2385R	GAGGGAGTTCTTCTTCTAGG	

To determine the lower detection limit of the nested PCR in this study, we carried out the PCR using 10-fold serially diluted template DNA, which was extracted from a sample with known concentration of HBV DNA with 2000 copies/ml. The DNA was extracted from 200 μl serum and dissolved in 200 μl TE.

To prevent carryover or cross contamination during the extraction of DNA from plasmas and PCR, each step of the procedure was performed in separate areas with dedicated equipment. Negative controls, including plasmas DNA from normal subjects without HBV infection and distilled water, and a positive control (an HBV DNA positive plasma diluted to 20 copies/ml) were always included in every nested PCR test.

### Quantitative assay of HBV DNA

Plasma HBV DNA in occult HBV infections was quantified by real-time PCR with a commercially available fluorescent real-time PCR assay (Shenyou Biotechnology, Shanghai, China) which had a strict internal quality control and passed the Clinical Laboratory Center of Chinese Ministry of Health external quality assessment on the DNA Engine Opticon 2 System (MJ Research, Waltham, MA). Plasma DNA was extracted using a commercial kit (Shenyou Biotechnology) from 20 μl plasma by boiling. The PCR was run for 40 cycles and the fluorescence signal of the amplicons was detected by the DNA Engine Opticon 2 System. The lower detection limit of this assay was 500 copies/ml with a linear range of up to 10^8 ^copies/ml.

### Detection of serological markers for HBV infection

Anti-HBc of the HBV DNA positive samples was measured by ELISA using the Diagnostic kit (Huakang Biotechnology, Shenzhen, China) for anti-HBc.

### HBV DNA sequencing and HBV genotyping

The PCR products amplified by S region primers were purified and directly sequenced on an ABI Prism 3130 sequencer (Applied Biosystems, Hitachi, Tokyo, Japan) after reaction with BigDye Terminator v3.1 (Applied Biosystems, Foster, CA). Sequence analysis and comparison were conducted by using molecular programs deposited in the web site of the National Centre for Biotechnology Information http://www.ncbi.nlm.nih.gov/. The sequences were compared with the same region of HBV sequences from different genotypes found in the genotyping reference set available on the NCBI website (URL: http://www.ncbi.nih.gov/projects/genotyping/view.cgi?db=2). The phylogenetic tree was constructed according to previous methods [47].

## Competing interests

The authors declare that they have no competing interests.

## Authors' contributions

YL and PL performed the experiments, analyzed the data, and drafted the manuscript and contributed equally to this work. CL collected the samples and assisted in the performance of the experiments. JZ carried out the DNA sequencing. CW interpreted the data and revised the manuscript. YHZ designed the study, interpreted the data and critically revised the manuscript. All authors read and approved the final manuscript.

## References

[B1] Gerlich WH Bremer C Saniewski M Schuttler CG Wend UC Willems WR Glebe D Occult hepatitis B virus infection: detection and significance Digest Dis 2010 28 116 125 10.1159/000282074 20460899

[B2] Raimondo G Allain JP Brunetto MR Buendia MA Chen DS Colombo M Craxi A Donato F Ferrari C Gaeta GB Gerlich WH Levrero M Locarnini S Michalak T Mondelli MU Pawlotsky JM Pollicino T Prati D Puoti M Samuel D Shouval D Smedile A Squadrito G Trepo C Villa E Will H Zanetti AR Zoulim F Statements from the Taormina expert meeting on occult hepatitis B virus infection J Hepatol 2008 49 652 657 10.1016/j.jhep.2008.07.014 18715666

[B3] Zhang R Wang L Li J Hepatitis B virus transfusion risk in China: proficiency testing for the detection of hepatitis B surface antigen Transfusion Med 2010 in press 10.1111/j.1365-3148.2010.01007.x 20409073

[B4] Niederhauser C Mansouri Taleghani B Graziani M Stolz M Tinguely C Schneider P Blood donor screening: how to decrease the risk of transfusion-transmitted hepatitis B virus? Swiss Med Wkly 2008 138 134 141 18330733 10.4414/smw.2008.12001

[B5] Calderon GM Gonzalez-Velazquez F Gonzalez-Bonilla CR Novelo-Garza B Terrazas JJ Martinez-Rodriguez ML Cortes-Marquez SR Blanco-Flores JP Rodriguez-Rodriguez A Del Campo MA Cortes-Gomez R Mejia-Bocanegra MG Prevalence and risk factors of hepatitis C virus, hepatitis B virus, and human immunodeficiency virus in multiply transfused recipients in Mexico Transfusion 2009 49 2200 2207 10.1111/j.1537-2995.2009.02248.x 19538543

[B6] Kafi-abad SA Rezvan H Abolghasemi H Talebian A Prevalence and trends of human immunodeficiency virus, hepatitis B virus, and hepatitis C virus among blood donors in Iran, 2004 through 2007 Transfusion 2009 49 2214 2220 10.1111/j.1537-2995.2009.02245.x 19527477

[B7] Hollinger FB Hepatitis B virus infection and transfusion medicine: science and the occult Transfusion 2008 48 1001 1026 10.1111/j.1537-2995.2008.01701.x 18454738

[B8] Giudice CL Martinengo M Pietrasanta P Bocciardo L Malavasi C Rastelli S Faraci M Tripodi G Occult hepatitis B virus infection: a case of reactivation in a patient receiving immunosuppressive treatment for allogeneic bone marrow transplantation Blood transfus 2008 6 46 50 18661923 10.2450/2008.0033-07 PMC2626849

[B9] Raimondo G Pollicino T Romano L Zanetti AR A 2010 update on occult hepatitis B infection Pathol Biol 2010 58 254 257 10.1016/j.patbio.2010.02.003 20303674

[B10] Liang X Bi S Yang W Wang L Cui G Cui F Zhang Y Liu J Gong X Chen Y Wang F Zheng H Wang F Guo J Jia Z Ma J Wang H Luo H Li L Jin S Hadler SC Wang Y Epidemiological serosurvey of hepatitis B in China--declining HBV prevalence due to hepatitis B vaccination Vaccine 2009 27 6550 6557 10.1016/j.vaccine.2009.08.048 19729084

[B11] Zhou YH Wu C Zhuang H Vaccination against hepatitis B: the Chinese experience Chin Med J (Engl) 2009 122 98 102 19187625

[B12] Zhang S Li RT Wang Y Liu Q Zhou YH Hu Y Seroprevalence of hepatitis B surface antigen among pregnant women in Jiangsu, China, 17 years after introduction of hepatitis B vaccine Int J Gynecol Obstet 2010 109 194 197 10.1016/j.ijgo.2010.01.002 20152977

[B13] Brechot C Thiers V Kremsdorf D Nalpas B Pol S Paterlini-Brechot P Persistent hepatitis B virus infection in subjects without hepatitis B surface antigen: clinically significant or purely "occult"? Hepatology 2001 34 194 203 10.1053/jhep.2001.25172 11431751

[B14] Zervou EK Dalekos GN Boumba DS Tsianos EV Value of anti-HBc screening of blood donors for prevention of HBV infection: results of a 3-year prospective study in Northwestern Greece Transfusion 2001 41 652 658 10.1046/j.1537-2995.2001.41050652.x 11346702

[B15] Shang G Seed CR Wang F Nie D Farrugia A Residual risk of transfusion-transmitted viral infections in Shenzhen, China, 2001 through 2004 Transfusion 2007 47 529 539 10.1111/j.1537-2995.2006.01146.x 17319836

[B16] Fang Y Shang QL Liu JY Li D Xu WZ Teng X Zhao HW Fu LJ Zhang FM Gu HX Prevalence of occult hepatitis B virus infection among hepatopathy patients and healthy people in China J Infect 2009 58 383 388 10.1016/j.jinf.2009.02.013 19329189

[B17] Shang G Yan Y Yang B Shao C Wang F Li Q Seed CR Two HBV DNA+/HBsAg-blood donors identified by HBV NAT in Shenzhen, China Transfus Apher Sci 2009 41 3 7 10.1016/j.transci.2009.05.001 19487161

[B18] Bamaga MS Azahar EI Al-Ghamdi AK Al-Enzi FQ Farahat FM Nucleic acid amplification technology for hepatitis B virus, and its role in blood donation screening in blood banks Saudi Med J 2009 30 1416 1421 19882053

[B19] Gonzalez R Torres P Castro E Barbolla L Candotti D Koppelman M Zaaijer HL Lelie N Allain JP Echevarria JM Efficacy of hepatitis B virus (HBV) DNA screening and characterization of acute and occult HBV infections among blood donors from Madrid, Spain Transfusion 2009 50 221 230 10.1111/j.1537-2995.2009.02343.x 19682332

[B20] Kwok S Higuchi R Avoiding false positives with PCR Nature 1989 339 237 238 10.1038/339237a0 2716852

[B21] Raimondo G Pollicino T Cacciola I Squadrito G Occult hepatitis B virus infection J Hepatol 2007 46 160 170 10.1016/j.jhep.2006.10.007 17112622

[B22] Lelie N Heaton A Hepatitis B - a review of the role of NAT in enhancing blood safety J Clin Virol 2006 36 Suppl 1 S1 2 10.1016/S1386-6532(06)80001-6 16831686

[B23] Offergeld R Faensen D Ritter S Hamouda O Human immunodeficiency virus, hepatitis C and hepatitis B infections among blood donors in Germany 2000-2002: risk of virus transmission and the impact of nucleic acid amplification testing Euro Surveill 2005 10 8 11 15735310

[B24] Chevrier MC St-Louis M Perreault J Caron B Castilloux C Laroche J Delage G Detection and characterization of hepatitis B virus of anti-hepatitis B core antigen-reactive blood donors in Quebec with an in-house nucleic acid testing assay Transfusion 2007 47 1794 1802 10.1111/j.1537-2995.2007.01394.x 17880603

[B25] Owusu-Ofori S Temple J Sarkodie F Anokwa M Candotti D Allain JP Predonation screening of blood donors with rapid tests: implementation and efficacy of a novel approach to blood safety in resource-poor settings Transfusion 2005 45 133 140 10.1111/j.1537-2995.2004.04279.x 15660820

[B26] Wang DW Wang TB Liu FP Shi LL Study about seroconversion of HBV NAT screening-positive crowd from blood donors Chinese Journal of Experimental and Clinical Virology 2008 22 127 129 18574536

[B27] Chen BY Shen J HBV-DNA detection in HBsAg negative blood donors and its clinical significance Chinese Journal of Nosocomiology 2007 10 1240 1241

[B28] Wang D Tan D Cao X A prospective study of posttransfusion hepatitis B virus infection Chinese Journal of Experimental and Clinical Virology 2000 14 77 79 11503032

[B29] Kreutz C Molecular, immunological and clinical properties of mutated hepatitis B viruses J Cell Mol Med 2002 6 113 143 10.1111/j.1582-4934.2002.tb00317.x 12003675 PMC6740305

[B30] Laulu SL Roberts WL The analytic sensitivity and mutant detection capability of six hepatitis B surface antigen assays Am J Clin Pathol 2006 125 748 751 10.1309/K5EM795VNGGFGBXX 16707377

[B31] Weber B Diagnostic impact of the genetic variability of the hepatitis B virus surface antigen gene J Med Virol 2006 78 Suppl 1 S59 65 10.1002/jmv.20610 16622880

[B32] Hollinger FB Hepatitis B virus genetic diversity and its impact on diagnostic assays J Viral Hepat 2007 14 Suppl 1 S11 15 10.1111/j.1365-2893.2007.00910.x 17958637

[B33] Chemin I Trepo C Clinical impact of occult HBV infections J Clin Virol 2005 34 Suppl 1 S15 21 10.1016/S1386-6532(05)80005-8 16461218

[B34] Gerlich WH Glebe D Schuttler CG Deficiencies in the standardization and sensitivity of diagnostic tests for hepatitis B virus J Viral Hepat 2007 14 Suppl 1 16 21 10.1111/j.1365-2893.2007.00912.x 17958638

[B35] Hollinger FB Sood G Occult hepatitis B virus infection: a covert operation J Viral Hepat 2010 17 1 15 10.1111/j.1365-2893.2009.01245.x 20002296

[B36] Zerbini A Pilli M Boni C Fisicaro P Penna A Di Vincenzo P Giuberti T Orlandini A Raffa G Pollicino T Raimondo G Ferrari C Missale G The characteristics of the cell-mediated immune response identify different profiles of occult hepatitis B virus infection Gastroenterology 2008 134 1470 1481 10.1053/j.gastro.2008.02.017 18355815

[B37] Torbenson M Thomas DL Occult hepatitis B Lancet Infect Dis 2002 2 479 486 10.1016/S1473-3099(02)00345-6 12150847

[B38] Komiya Y Katayama K Yugi H Mizui M Matsukura H Tomoguri T Miyakawa Y Tabuchi A Tanaka J Yoshizawa H Minimum infectious dose of hepatitis B virus in chimpanzees and difference in the dynamics of viremia between genotype A and genotype C Transfusion 2008 48 286 294 18028278 10.1111/j.1537-2995.2007.01522.x

[B39] Candotti D Allain JP Transfusion-transmitted hepatitis B virus infection J Hepatol 2009 51 798 809 10.1016/j.jhep.2009.05.020 19615780

[B40] Hoofnagle JH Waggoner JG Hepatitis A and B virus markers in immune serum globulin Gastroenterology 1980 78 259 263 6243109

[B41] Levicnik-Stezinar S Rahne-Potokar U Candotti D Lelie N Allain JP Anti-HBs positive occult hepatitis B virus carrier blood infectious in two transfusion recipients J Hepatol 2008 48 1022 1025 10.1016/j.jhep.2008.02.016 18436328

[B42] Wendel S Levi JE Biagini S Candotti D Allain JP A probable case of hepatitis B virus transfusion transmission revealed after a 13-month-long window period Transfusion 2008 48 1602 1608 10.1111/j.1537-2995.2008.01723.x 18466175

[B43] Jackson BR Busch MP Stramer SL AuBuchon JP The cost-effectiveness of NAT for HIV, HCV, and HBV in whole-blood donations Transfusion 2003 43 721 729 10.1046/j.1537-2995.2003.00392.x 12757522

[B44] Toyoda H Hayashi K Murakami Y Honda T Katano Y Nakano I Goto H Kumada T Takamatsu J Prevalence and clinical implications of occult hepatitis B viral infection in hemophilia patients in Japan J Med Virol 2004 73 195 199 10.1002/jmv.20075 15122792

[B45] Hui CK Sun J Au WY Lie AK Yueng YH Zhang HY Lee NP Hou JL Liang R Lau GK Occult hepatitis B virus infection in hematopoietic stem cell donors in a hepatitis B virus endemic area J Hepatol 2005 42 813 819 10.1016/j.jhep.2005.01.018 15885351

[B46] Kim SM Lee KS Park CJ Lee JY Kim KH Park JY Lee JH Kim HY Yoo JY Jang MK Prevalence of occult HBV infection among subjects with normal serum ALT levels in Korea J Infect 2007 54 185 191 10.1016/j.jinf.2006.02.002 16564573

[B47] Utama A Octavia TI Dhenni R Miskad UA Yusuf I Tai S Hepatitis B virus genotypes/subgenotypes in voluntary blood donors in Makassar, South Sulawesi, Indonesia Virol J 2009 6 128 10.1186/1743-422X-6-128 19691824 PMC2732614

